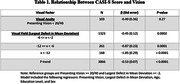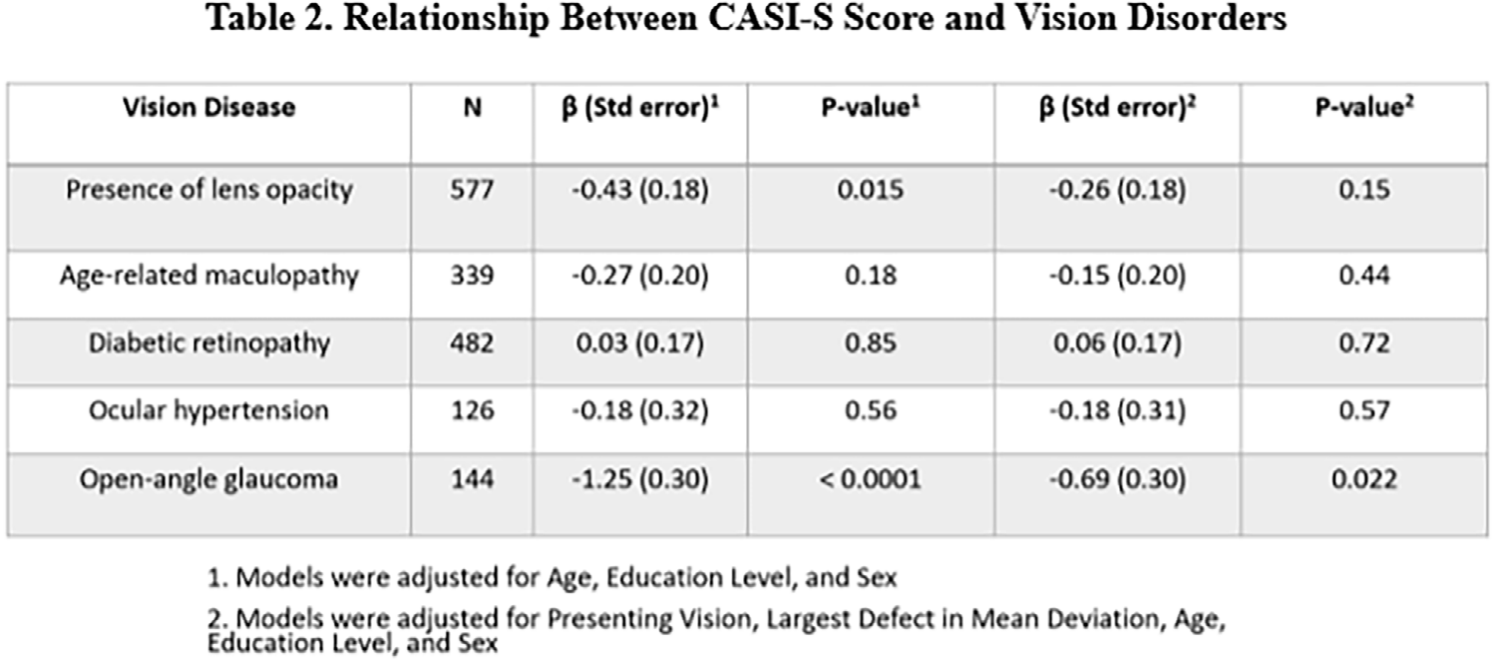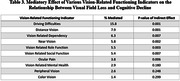# Driving Difficulty is an Important Mediator of the Impact of Visual Field Loss on Cognitive Impairment among Older Latinos

**DOI:** 10.1002/alz.094744

**Published:** 2025-01-09

**Authors:** Alan C Zheng, Roberta McKean‐Cowdin, Mina Torres, Rohit Varma, Xuejuan Jiang

**Affiliations:** ^1^ USC Roski Eye Institute, Keck School of Medicine of the University of Southern California, Los Angeles, CA USA; ^2^ Keck School of Medicine of the University of Southern California, Los Angeles, CA USA; ^3^ Southern California Eye Institute, CHA Hollywood Presbyterian Medical Center, Los Angeles, CA USA

## Abstract

**Background:**

Recent studies have identified visual impairment as a potentially modifiable dementia risk factor. However, the underlying mechanism linking visual impairment and cognitive decline is still not fully understood. Additionally, there has been a dearth of research focusing on Latinos.

**Method:**

Data were obtained from the Los Angeles Latino Eye Study (LALES), a population‐based longitudinal study of Mexican Americans aged 40 and above. The study performed comprehensive eye examination. Self‐reported vision‐related functioning was assessed using the National Eye Institute Visual Function Questionnaire (NEI‐VFQ‐25). Vision data were collected from the baseline visit, while the cognition data were obtained from the 8‐year follow up visit using the Cognitive Abilities Screening Instrument‐Short (CASI‐S) form. Multivariable linear regression models were used to assess the association with cognition. We explored the mediation effect of vision‐related health domains, such as driving difficulty, dependency, social function, and mental health. All analyses were conducted in R program.

**Result:**

A total of 3904 participants had valid vision and cognition data available for analyses. Visual acuity was not associated with CASI‐S score (P = 0.27) after adjusting for age, education level, and sex. However, greater visual field loss at baseline had a dose‐dependent association with lower CASI‐S score (P trend<0.001). Compared with individuals without visual field loss (mean deviation of ≥‐2 decibels [dB]), those with mild (‐6 dB ≤ to ←2dB), moderate (‐12dB ≤ to ←6dB), and severe (←12dB) loss demonstrated CASI‐S scores 0.45, 0.87, and 1.85 lower, respectively (Ps<0.001). With regards to individual vision disorders, such as cataracts, age‐related maculopathy, and diabetic retinopathy, no significant associations with CASI‐S scores were observed after adjusting for visual field loss, except for open‐angle glaucoma (β = ‐0.69, P = 0.022). Of the 10 vision‐related functioning indicators tested, driving difficulty had the largest mediatory effect. Notably, vision‐related driving difficulty was found to mediate 15.8% of the relationship between visual field loss and CASI‐S score, and social functioning accounted for 5.4%.

**Conclusion:**

In a cohort of community‐dwelling older Latinos, we found that visual field loss is associated with cognitive impairment. Vision‐related driving difficulty was identified as a more important mediator than social functioning, dependency, and mental health.